# Supporting the strategic pillars of translational research in biofilms

**DOI:** 10.1038/s41522-022-00354-4

**Published:** 2022-11-13

**Authors:** Miguel Camara, Alain Filloux

**Affiliations:** 1 National Biofilms Innovation Centre, Biodiscovery Institute and School of Life Sciences, University of Nottingham, Nottingham, UK; 2 Singapore Centre for Environmental Life Sciences Engineering, Nanyang Technological University, Singapore, Singapore

**Keywords:** Biofilms, Microbial ecology, Clinical microbiology, Environmental microbiology, Bioremediation

It is now widely accepted that most microorganisms live within communities and not as single cells. The interaction between species within the community drives the behaviour of the population and its adaptation to various ecosystems and hosts. This relatively recent concept has triggered the emergence of a whole research field in Microbiology aiming at understanding the structure and organisation of bacterial biofilms combined with the detail of microbiome composition and evolution. Researchers also joined forces in what is a multidisciplinary field of investigation calling for knowledge from basic microbiology to surface chemistry and all the way to advanced omics and microscopy methodologies. The National Biofilms Innovation Centre was launched in 2017 to address the impact of biofilms on global challenges from antimicrobial resistance and food safety to water security by bringing together UK research and businesses to drive the translation of biofilm research into innovative solutions. Since its creation, NBIC has built a national partnership with 63 UK research institutions and more than 250 companies and an international partnership with biofilms centres across the world including the Centre for Biofilm Engineering (CBE) in the United States (Montana) and the Singapore Centre for Environmental Life Sciences Engineering (SCELSE).

This special collection in *npj Biofilms and Microbiomes* has been created to review the impact of biofilm across sectors, illustrate some translational research on biofilm carried out by NBIC partners and highlight key issues on the successful translation of biofilm research. The collection addresses the four interventional pillars of NBIC: prevent, detect, manage and engineer.

Biofilms have an economic impact of 5Tn USD ($) per year across multiple sectors, as detailed in perspective by Camara et al.^1^, with biofilms being central to some of the most important global challenges. Addressing these challenges through the above interventional pillars opens endless opportunities for growth by harnessing and controlling the biological activities of complex biofilms.

The *Prevent theme* focuses on the prevention of early stage microbial adhesion and colonisation events at surfaces and interfaces and curtailing the development and maturation of early stage biofilms. The two main approaches used to prevent biofilm formation are through early microbial killing or preventing the establishment of the biofilm in the first place. This collection shows two examples of these approaches. The work from Barbieri et al.^2^ uses a simple way to functionalise a common silicon-based polymer utilised in biomedical and industrial applications for the slow release of antimicrobials already approved for human use. This modified polymer can drastically reduce the ability of bacteria to colonise these surfaces providing a scalable new alternative for the functionalisation of catheters, dressings and in-dwelling medical devices to existing strategies, such as the in situ generation of silver nanoparticles^3^. In another study, Pentland et al.^4^ show that *C. albicans* uses CO_2_ found in the host environment to promote its ability to colonise and compete for nutrients. These findings reveal that the design of therapeutic interventions targeting CO_2_ utilisation could be used as a new approach to prevent *C. albicans* biofilm formation in high CO_2_ environments paving the way for new therapeutic approaches against infections caused by this organism.

The *Detect theme* focuses on the requirement for accurate, quantitative biofilm detection and also measurements across multiple scales through innovative sensing, tracking and diagnostic technologies. The detection and quantification of morphological changes in a biofilm is frequently done using confocal microscopy. However, there have been issues with regards to the reproducibility of image analysis across laboratories due to some of them not reporting the full exact imaging methods and parameters used. This is essential when biofilm interventions are compared across laboratories using techniques such as LIVE/DEAD® staining. Mountcastle et al.^5^ have developed an automated image analysis approach which has shown reproducible and robust results in a range of bacterial species biomass and cell viability measurements. This method is highly accessible to researchers from different disciplines supporting their biofilm translational studies. There is also a need not only to visualise and quantify biofilms but also to develop novel technologies which enable scientists to visualise changes in a biofilm in real time such as viability and metabolic changes. A study by Wang et al.^6^ shows the use of resonant hyperspectral imaging to monitor in real-time bacterial cell attachment and micro-colony formation. With this technique the authors were able to focus on the underside of the biofilm providing a very clear picture of the early stages of biofilm formation and the fate of biofilms in response to antibiotics treatment. Another study by Hollmann et al.^7^ show the development of ratiometric fluorescent pH-sensitive nanosensors which enable the visualisation of pH changes in biofilms. Here the authors demonstrated that these nanosensors could be used to detect sugar metabolism in real time. The use of alternative dyes in these nanosensors will generate powerful tools to monitor wider microniche changes within biofilms at high resolution.

The mission of the *Manage theme* is to kill, remove or control established biofilms by exploiting the knowledge gained from understanding the mechanisms governing biofilm life cycle dynamics and development, their physicochemical properties, and levels of complexity across a range of environments. Biofilms can adapt quickly to changing environmental conditions and rapidly select for mutants that increase their fitness. This poses a serious issue in the clinic with the adaptation of biofilms to antibiotic use. Some of these biofilms, and the cells sitting within different regions of such an environmentally heterogenous structure, are frequently exposed to sub-inhibitory concentrations of antibiotics yet, there is limited understanding of how they adapt to these levels of antibiotics. Trampari et al.^8^ show that biofilms from *Salmonella* Typhimurium exposed to sub-inhibitory concentrations of three antibiotics rapidly evolve resistance to them. This adaptation resulted in a deficit in biofilm formation but strains forming the least biofilms were more virulent in a *Galleria mellonella* infection model resulting in the lowest survival rates. This is supporting both the concept of lifestyle switch and trade-off when gaining novel function/feature. The resilience of biofilms to the action of antibiotics has also led to a search for novel approaches to eradicate them. One of these approaches is based on the use of phage components such as endolysins. In a study by Arroyo-Moreno et al.^9^ the authors show that endolysins can disrupt *Gardnerella vaginalis* both in single species and polymicrobial biofilms with no effect on microorganisms from the commensal vaginal flora nor the emergence of resistance, offering a promising strategy to combat bacterial vaginosis. An alternative approach to identify novel antimicrobials is to search from them in microbiomes as they are a great resource for bioactive molecules which has been very much unexploited. In the study by Mulkern et al.^10^ the authors used antimicrobial peptides form rumen microbiome that show high effectiveness against *Pseudomonas aeruginosa* biofilms with low cytotoxicity and hence therapeutic potential. Finally, when designing new antimicrobials, it is important to understand their impact on different components of the biofilm such as the extracellular matrix, especially if the drug interacts with it like polymyxin B. One of the most common ways to visualise changes in this matrix requires the use of dyes which can frequently alter its physicochemical properties preventing an accurate visualisation of the changes. To overcome this limitation, Powel et al.^11^ have used multiple particle tracking (MPT) to characterise the physical and mechanical properties of antimicrobial resistant (AMR) bacterial biofilms and quantify the effects of antibiotic treatment. Using nanoparticles (NPs) of varying charge and size they were able to quantify the diffusion and mechanical effects of antibiotic therapies within the AMR biofilm matrix as a result of changes in the viscoelasticity of the biofilm. The authors found that this approach offers a valuable tool for the pre-clinical screening of new antibiofilms.

The vision of the *Engineer theme* is the control of biofilms in industrial environments and large-scale infrastructure, and the engineering of bespoke biofilms for targeted applications.

Engineering biofilm communities for more effective anaerobic digestion has great potential in organic/wastewater treatment as it offers the opportunity to recover biogas as energy whilst reducing risks to human health and the environment. For the engineering of these systems the core microorganisms have traditionally been considered. However, the study by Guo et al.^12^ reveals that microbes in lower abundance could have a key role in anaerobic digestion maintaining the stability and functionality of microbial communities. Therefore, the ecological role of low-abundance taxa should be further investigated in bioreactors with significant environmental impact. Biofilms can also be engineered to reduce the impact of global warming by contributing to the reduction of CO_2_ levels. A range of approaches, including electrochemical methods, have been developed to reduce CO_2_ to valuable chemicals. As such, microbial electrosynthesis (MES) offers an alternative for the sustainable conversion of CO_2_ into valuable organic chemicals at the cathode of bio-electrochemical systems where microorganisms are used as biocatalysts. A major bottleneck with MES lies with the development and scale up of the technology due to the slow development of CO_2_-reducing biofilms on the cathodes. Izadi et al.^13^ showed a strategy which can potentially bypass this issue by modifying the potential applied and the inorganic carbon source used as these can enhance the efficiency of the system by affecting the development of the biofilm during MES and its microbial composition. Keeping biofilms under control in drinking water systems is also paramount to maintain water quality standards. Phosphate dosing has been regularly used to prevent plumbosolvency in water supply networks; however, the impact this may have on polymicrobial communities in these networks had not been studied. Del Olmo et al.^14^ have studied the effect of phosphate on the growth of polymicrobial biofilms from drinking water grown on lead and PVC. They found that changes in phosphate levels can induce changes in the structure of biofilms and affect the level of chlorine in the water. Their studies will help improve the management strategies to control microbial growth in drinking water pipelines helping to increase its quality.

For all the above findings to be successfully translated from the academic research lab into the industrial environment it is paramount to have a cross-sectorial engagement at the conception of the technology to facilitate its evolution along a Translationally Optimal Path (TOP). This is highlighted by Highmore et al.^15^ who have created a two-dimensional framework they have termed the Biofilm Research-Industrial Engagement Framework (BRIEF). This framework enables the organisation of current biofilm technologies relevant to biofilms across sectors, based on their accuracy to current science knowledge and industrial application, enabling to predict the chances of evolving through Technology Readiness Levels (TRLs). They also introduce some advisory guidelines to enhance the translational impact of future biofilm research.
